# Prognostic value of IVIM-DWI parameters for overall and recurrence-free survival in resected esophageal squamous cell carcinoma

**DOI:** 10.1186/s40644-026-01077-x

**Published:** 2026-06-25

**Authors:** Yifan Hu, Jiaye Pan, Qian Xia, Xin Han, Zeyu Tang, Jin Chen, Lin Kong

**Affiliations:** 1 Department of Radiology, Dongtai People’s Hospital, 2# Kangfuxi Rd, Dongtai, Jiangsu Province 224200 China; 2 Department of Ultrasound, Dongtai Second People’s Hospital, Dongtai, Jiangsu Province China; 3 Department of Thoracic Surgery, Dongtai People’s Hospital, 2# Kangfuxi Rd, Yancheng, China

**Keywords:** Esophageal squamous cell carcinoma, Intravoxel incoherent motion, Apparent diffusion coefficient, Overall survival, Recurrence-free survival

## Abstract

**Purpose:**

Prognostic stratification after surgical resection remains challenging in esophageal squamous cell carcinoma (ESCC), as pathological staging cannot quantitatively capture intratumoral heterogeneity. Intravoxel incoherent motion diffusion-weighted imaging (IVIM-DWI) enables noninvasive quantification of tumor diffusion and perfusion characteristics. To evaluate the prognostic value of IVIM-derived parameters for predicting overall survival (OS) and recurrence-free survival (RFS) in resected ESCC and to compare their performance with conventional clinicopathological factors.

**Methods:**

Eighty patients with pathologically confirmed ESCC who underwent preoperative IVIM-DWI MRI and surgical resection were retrospectively included. IVIM parameters (apparent diffusion coefficient (ADC), the true diffusion coefficient (D), pseudo-diffusion coefficient (D*), and perfusion fraction (f)) were extracted using maximum-diameter and whole-volume tumor delineation. Cox regression with stepwise selection was performed to identify prognostic factors. Time-dependent ROC analysis was used to evaluate the predictive performance of clinicopathological, IVIM-based, and combined models, and Kaplan–Meier survival analysis was applied to assess risk stratification based on model-derived risk scores.

**Results:**

IVIM-based models showed superior prognostic performance compared with clinicopathological models, particularly for OS. For 1-year OS prediction, the IVIM model (maximum-diameter) achieved an AUC of 0.776 and an accuracy of 0.725, while the combined model further improved performance (AUC 0.827, accuracy 0.812). Similar advantages were observed for RFS, with the combined model yielding a 1-year AUC of 0.825 and accuracy of 0.812, and maintaining the best performance at 3 years for both OS (AUC 0.719, accuracy 0.738) and RFS (AUC 0.850, accuracy 0.825). In addition, the derived risk scores enabled effective risk stratification, with Kaplan–Meier analyses demonstrating significant survival differences between risk groups (log-rank *P* < 0.001).

**Conclusion:**

IVIM-derived parameters provide complementary prognostic information beyond conventional pathological staging in ESCC and may improve postoperative risk stratification when integrated with clinicopathological factors.

**Supplementary Information:**

The online version contains supplementary material available at https://doi.org/10.1186/s40644-026-01077-x.

## Introduction

Esophageal cancer (EC) is a leading malignancy, ranking sixth in cancer-related mortality [1]. Despite advances in surgical techniques, radiotherapy, and systemic therapies, the long-term prognosis of patients with esophageal cancer remains unsatisfactory, with high rates of recurrence and poor overall survival (OS). Accurate risk stratification and individualized prognostic assessment are therefore essential to guide treatment decisions and postoperative surveillance strategies [2].

Currently, tumor–node–metastasis (TNM) staging, based on postoperative pathological evaluation, is the cornerstone for prognostic assessment in esophageal cancer. Pathological T stage reflects tumor invasion depth, while nodal status remains one of the most powerful predictors of recurrence and survival [3, 4]. However, patients with identical TNM stages often exhibit markedly heterogeneous clinical outcomes, suggesting that conventional pathological staging alone may be insufficient to fully capture the biological aggressiveness of esophageal tumors. This limitation has prompted increasing interest in imaging biomarkers that can noninvasively characterize tumor microstructure and biological behavior beyond anatomical staging [5].

Diffusion-weighted imaging (DWI) has emerged as a valuable functional magnetic resonance imaging (MRI) technique for tumor characterization by probing the diffusion of water molecules within tissues [6, 7]. Intravoxel incoherent motion (IVIM) imaging extends conventional DWI by separating pure molecular diffusion from microcirculatory perfusion-related diffusion using a bi-exponential model. IVIM-derived parameters, including the true diffusion coefficient (D), pseudo-diffusion coefficient (D*), and perfusion fraction (f), provide complementary information regarding tumor cellularity, microvascular perfusion, and tissue heterogeneity [8]. Increasing evidence suggests that IVIM parameters are associated with tumor grade, angiogenesis, hypoxia, and treatment response in various malignancies [9, 10].

In esophageal cancer, previous IVIM studies have mainly investigated its value for tumor characterization, staging, lymph node evaluation, and treatment-response assessment [11, 12]; for example, Liu et al. used IVIM parameters to differentiate pathological grades of esophageal squamous cell carcinoma (ESCC), while Song et al. evaluated their value for preoperative T staging and lymph node metastasis prediction [13, 14]. However, these studies largely focused on diagnostic or short-term response endpoints, and the prognostic significance of IVIM parameters for long-term outcomes, such as recurrence-free survival (RFS) and OS, remains incompletely understood. Moreover, few studies have systematically compared the predictive performance of IVIM parameters with that of established clinicopathological factors, particularly TNM staging, in a head-to-head manner.

Given the multifactorial nature of tumor progression and recurrence, conventional clinicopathological factors may not fully capture the biological heterogeneity underlying postoperative outcomes [15]. Functional imaging biomarkers may therefore provide complementary information for prognostic assessment. Among these approaches, IVIM has the advantage of providing a limited number of physiologically interpretable quantitative parameters reflecting molecular diffusion and microcirculatory perfusion, whereas some advanced MRI or radiomics approaches may involve more complex acquisition protocols, contrast administration, or high-dimensional feature extraction [8, 16]. However, it remains unclear whether IVIM parameters provide incremental prognostic value over traditional TNM staging, or whether a combined model incorporating both pathological and IVIM-derived features yields superior predictive performance for survival outcomes in patients with esophageal cancer.

Therefore, this study aimed to compare the prognostic value of pathological TNM-related variables and IVIM parameters derived from maximum-diameter and whole-volume analyses for predicting RFS and OS in patients with esophageal cancer. Unlike previous IVIM studies mainly focused on tumor characterization, staging, or treatment-response assessment, we further evaluated whether IVIM-derived features provide incremental prognostic value beyond pathological staging and improve postoperative risk stratification when integrated with clinicopathological factors.

## Materials and methods

### Study population

**Fig. 1:**
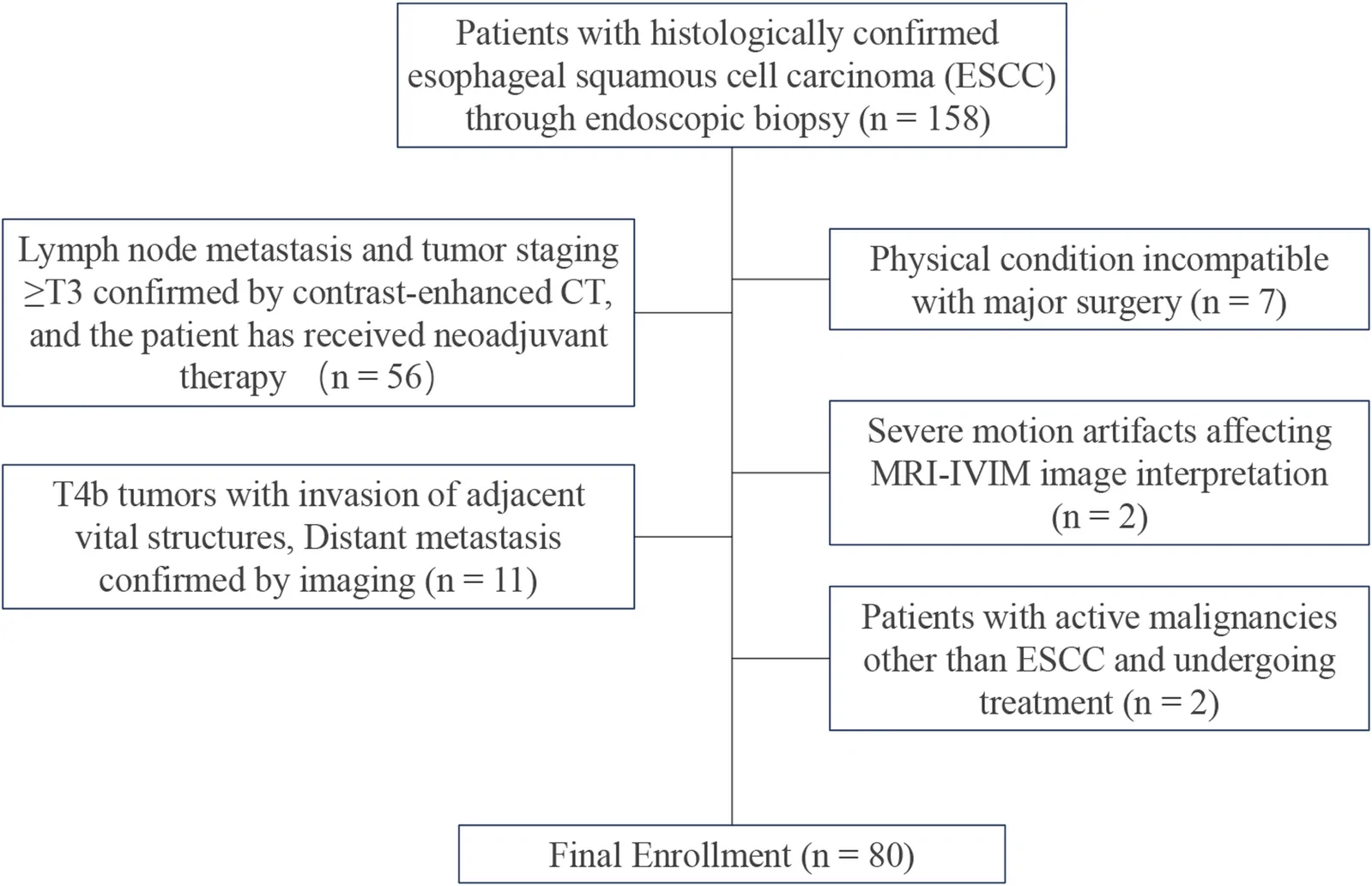
Flow chart of patient enrollment. This flow chart was adapted from our previously published study [17]

This study was approved by the Ethics Committee of Dongtai People’s Hospital (Approval No.: 2020-dtry-K-16) and conducted in accordance with the principles of the Declaration of Helsinki. Written informed consent was obtained from all participants. From January 2020 to September 2022, 158 consecutive patients with endoscopically confirmed ESCC were initially considered. The inclusion criteria were as follows: (1) histopathologically confirmed ESCC and (2) planned surgical resection with preoperative IVIM-DWI performed within 2 weeks before surgery. After screening, 78 patients were excluded for various reasons, leaving 80 patients who underwent both MRI evaluation and surgical treatment. The patient selection process is shown in Fig. 1 [17].

### MRI protocol

All MRI examinations were performed on a 3.0T scanner (Discovery MR 750 W, GE Medical Systems, Waukesha, WI, USA) using a 32-channel body coil. Patients fasted for at least 6 h and were instructed to maintain shallow breathing during the scan. The IVIM-DWI sequence incorporated shimming, respiratory triggering, and fat-suppression techniques to reduce motion artifacts. Acquisition parameters included repetition time ≈ 3000 ms, minimal echo time, slice thickness of 4.4 mm, inter-slice gap of 1.0 mm, FOV of 42 × 42 cm, and a 128 × 128 matrix. Eleven b-values (0, 20, 50, 80, 100, 150, 200, 400, 600, 800, and 1000 s/mm²) were applied, with corresponding excitations of 2, 2, 2, 2, 2, 2, 2, 3, 3, 4, and 6, respectively, resulting in a total IVIM-DWI scan time of approximately 6 min. Additional sequences included T1-weighted, T2-weighted, and fat-suppressed T2-weighted imaging.

### Imaging analysis

IVIM data were processed using Image Engine software (Vusion Tech) and independently evaluated by two experienced radiologists (with 9 and 15 years of experience, respectively) who were blinded to the pathological findings. Apparent diffusion coefficient (ADC) maps were generated using a mono-exponential model applied to 11 b-values, following the equation:

$$\:S\left(b\right)={S}_{0}\cdot\:{e}^{-b\cdot\:ADC}$$

where S(b) is the signal intensity at a given b-value, 𝑆_0_ is the signal intensity without diffusion weighting (b = 0).

For the estimation of true diffusion (D, also called ADC_slow), pseudo-diffusion (D*, also called ADC_fast), and perfusion fraction (f), a bi-exponential IVIM model was used:

$$\:S\left(b\right)={S}_{0}\cdot\:\left[\right(1-f)\cdot\:{e}^{-b\cdot\:D}+f\cdot\:{e}^{-b\cdot\:(D+D*)}]$$

Here, 𝑓 denotes the fraction of the fast-diffusion component, D represents true molecular diffusion, and D* corresponds to pseudo-diffusion reflecting microcirculatory perfusion.

**Fig. 2:**
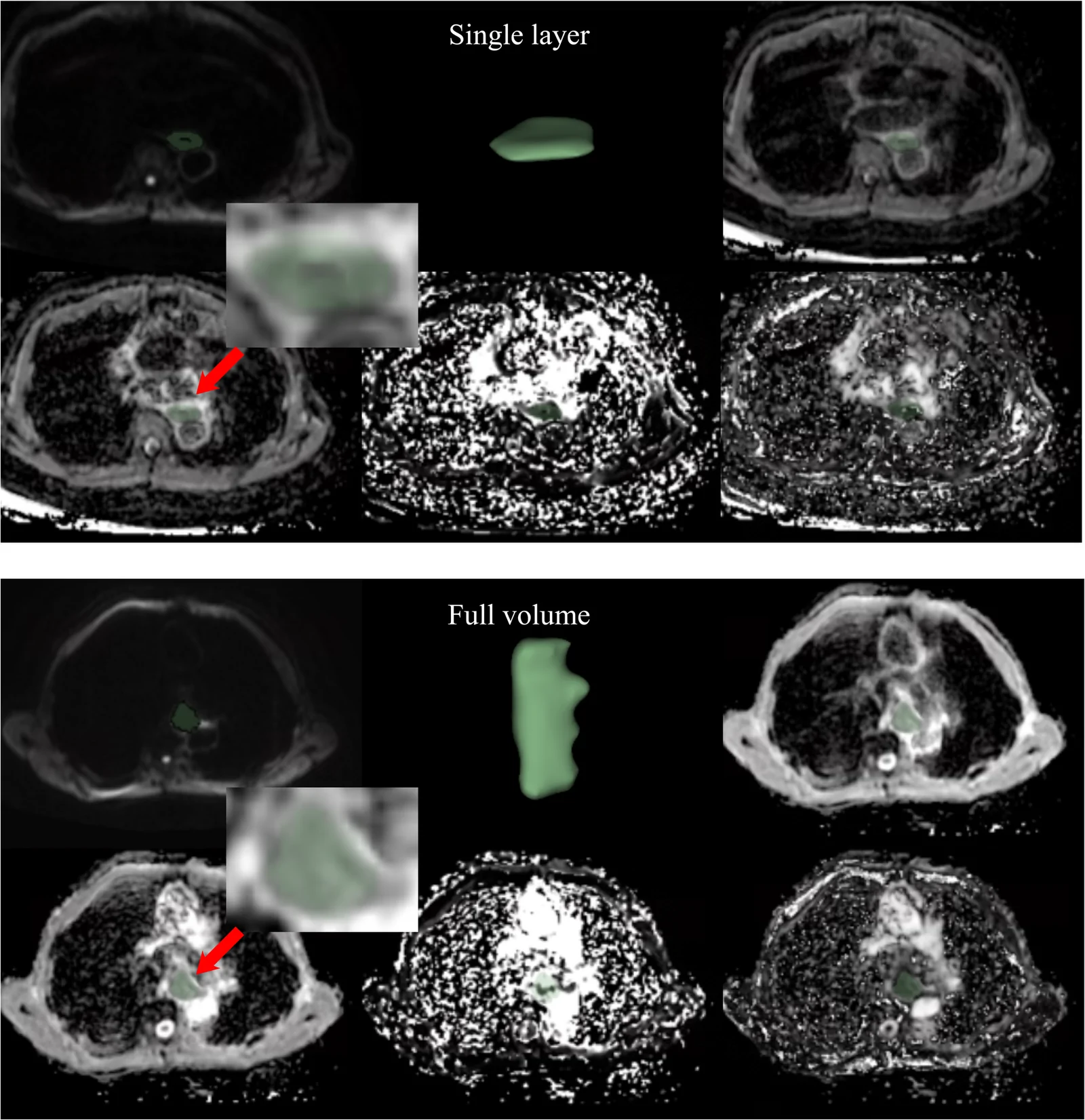
Illustration of maximum-diameter slice and whole-volume tumor delineation. This figure was adapted from our previous publication [17]

For each patient, the two radiologists manually delineated regions of interest (ROIs) on the largest cross-sectional area of the tumor’s solid components, followed by measurement (Fig. 2). Subsequently, the entire tumor volume was segmented for volumetric analysis. Care was taken to include as much of the solid tumor as possible while avoiding areas of hemorrhage, calcification, or necrosis, as evident on ADC, D, D*, and f maps. Mean, standard deviation (SD), maximum, and minimum values for ADC, D, D*, and f were calculated for both single-section and whole-volume ROIs. Interobserver agreement was assessed using the intraclass correlation coefficient (ICC) with the ICC (3, k) model.

### Pathological examination

Surgically resected specimens were staged according to the 8th edition of the American Joint Committee on Cancer (AJCC) TNM system for esophageal cancer. Tumor depth (T stage) was evaluated by a pathologist with 15 years of experience who was blinded to the imaging data. Patients were grouped according to esophageal wall invasion as T1–T2 versus T3–T4a. Lymph node status was classified as negative (N−) or positive (N+) based on the presence or absence of metastatic involvement. Lymphovascular invasion (LVI) was assessed on hematoxylin–eosin–stained sections and recorded as present or absent.

### Parameter selection

Clinical variables and IVIM-derived parameters were used to construct prognostic models for OS and RFS. IVIM parameters were extracted separately from a maximum-diameter slice (IVIM1) and from the whole-volume segmentation (IVIM2).

For clinical variables, multicollinearity was first assessed using the variance inflation factor (VIF). Variables with excessive collinearity were iteratively removed until all retained clinical variables showed acceptable collinearity, using a VIF threshold of 3. For IVIM-derived parameters, pairwise correlations were calculated before multivariable analysis. Highly correlated IVIM parameters were removed using a correlation threshold of 0.80 to reduce redundancy among imaging-derived variables. After collinearity assessment, the remaining clinical variables and IVIM parameters were entered separately into multivariable Cox proportional hazards regression models for OS and RFS. A bidirectional stepwise selection procedure based on the Akaike information criterion (AIC) was then applied to identify candidate predictors for subsequent prognostic model construction. Hazard ratios (HRs) and corresponding 95% confidence intervals (CIs) were calculated for the variables retained after stepwise selection.

### Time-dependent ROC analysis

Three types of models were constructed for OS and RFS prediction: (1) a clinical model including selected clinicopathological factors; (2) IVIM-based models constructed separately using IVIM1 and IVIM2 parameters; (3) combined models integrating clinical variables with IVIM parameters from IVIM1 (Combined1) or IVIM2 (Combined2).

For each model, a Cox proportional hazards regression model was fitted using the variables retained after feature selection, and the resulting linear predictor was used as the prognostic risk score. Model discrimination for OS and RFS was assessed at 12 and 36 months using time-dependent receiver operating characteristic (ROC) curve analysis, and the area under the ROC curve (AUC) was used to quantify predictive performance. To internally validate model performance and reduce overfitting concerns, 1000 bootstrap resamples were performed to calculate bootstrap-derived 95% CIs and optimism-corrected AUCs. The optimism-corrected AUC was calculated by subtracting the estimated bootstrap optimism from the apparent AUC. The models were compared to assess whether IVIM-derived parameters provided incremental prognostic value beyond clinicopathological variables. All statistical analyses were conducted using R software.

### Follow-up

All patients were followed up after surgical resection according to institutional standard protocols. Follow-up evaluations were conducted through outpatient visits, inpatient medical record review, and telephone interviews. Routine follow-up assessments included physical examination, laboratory tests, and imaging examinations such as contrast-enhanced computed tomography or magnetic resonance imaging, as clinically indicated.

Follow-up was performed every 3–6 months during the first two years after surgery and every 6–12 months thereafter. Recurrence was defined as radiological or pathological evidence of locoregional recurrence or distant metastasis. The date of recurrence was recorded as the time when recurrent disease was first confirmed by imaging or histopathological examination.

OS was defined as the interval between the date of surgery and the date of death from any cause or the last follow-up. RFS was defined as the interval between the date of surgery and the date of the first documented recurrence or death, whichever occurred first. Patients without recurrence or death were censored at the date of the last follow-up.

The follow-up period ended in November 2025. All survival and recurrence data were collected independently by two investigators who were blinded to the IVIM-derived parameters.

### Statistical analysis

The study data were analyzed using R software (version 4.4.2). Continuous variables were presented as mean ± standard deviation (¯x ± s) if they conformed to a normal distribution (determined by the Shapiro-Wilk test), or as median (interquartile range) if they did not. T-tests or Mann-Whitney U tests were employed to assess differences between groups. Parameters with an ICC exceeding 0.7 were incorporated into a stepwise regression analysis.

Risk stratification was performed based on the Cox proportional hazards models established for OS and RFS. For each patient, a risk score was calculated using the linear predictor derived from the Cox model.

Patients were stratified into high-risk and low-risk groups according to the median value of the risk score in the entire cohort. Kaplan–Meier survival curves were generated to evaluate OS and RFS for different risk groups, and differences between groups were compared using the log-rank test. A two-sided P value < 0.05 was considered statistically significant.

## Results

### Study population

A total of 80 patients were included in this study, with a median age of 70 years (Q1: 66; Q3: 74). The cohort comprised 61 males (76.3%) and 19 females (23.7%). At the last follow-up, 48 patients (60.0%) were alive and 32 patients (40.0%) had died. Regarding recurrence status, 29 patients (36.3%) remained recurrence-free, whereas 51 patients (63.7%) experienced disease recurrence. The median follow-up duration was 40.5 months. During follow-up, 10 deaths and 14 recurrence events were observed within the first year, increasing to 26 deaths and 27 recurrence events at 3 years. Baseline clinicopathological characteristics stratified by OS and RFS are summarized in Table 1.

**Table 1:** Baseline clinicopathological characteristics according to survival and recurrence status

Parameter	Overall Survival			Recurrence-Free Survival		
	Alive (*n* = 48)	Death (*n* = 32)	*P*	No recurrence (*n* = 29)	Recurrence (*n* = 51)	*P*
Gender (man)	36 (75.0%)	25 (78.1%)	0.795	25 (86.2%)	36 (70.6%)	0.172
Age	70 (65, 74)	71 (67, 74)	0.475	67 (63, 73)	72 (67, 74.5)	0.060
Smoking	24 (50%)	14 (43.8%)	0.651	16 (55.2%)	22 (43.1%)	0.356
Drinking	22 (45.8%)	15 (46.9%)	0.999	15 (51.7%)	22 (43.1%)	0.492
Location						
Up	6 (12.5%)	3 (9.4%)	0.470	2 (6.9%)	8 (15.7%)	0.800
Middle	26 (54.2%)	14 (43.8%)		16 (55.2%)	39 (76.5%)	
Low	16 (33.3%)	15 (46.9%)		11 (37.9%)	4 (7.8%)	
Volume (cm^3^)	1.50 (0.75, 2.04)	1.56 (1.33, 2.63)	0.118	1.47 (1.17, 2.60)	1.54 (0.83, 2.05)	0.638
N Staging						
-	31 (64.6%)	12 (37.5%)	0.023	12 (41.4%)	31 (60.8%)	0.108
+	17 (35.4%)	20 (62.5%)		17 (58.6%)	20 (39.2%)	
T Staging						
1–2	30 (62.5%)	15 (46.9%)	0.178	11 (37.9%)	34 (66.7%)	0.019
3-4a	18 (37.5%)	17 (53.1%)		18 (62.1%)	17 (33.3%)	
LVI						
-	31 (64.6%)	19 (59.4%)	0.646	18 (62.1%)	32 (62.7%)	1.000
+	17 (35.4%)	13 (40.6%)		11 (37.9%)	19 (37.3%)	
Histological Differentiation						
Well differentiated	8 (16.7%)	2 (6.2%)	0.146	2 (6.9%)	8 (15.7%)	0.004
Moderately differentiated	34 (70.8%)	21 (65.6%)		16 (55.2%)	39 (76.5%)	
Poorly differentiated	6 (12.5%)	9 (28.1%)		11 (37.9%)	4 (7.8%)	

LVI = lymphovascular invasion; Data are presented as n (%) or median (interquartile range). P values compare the corresponding survival or recurrence groups and were calculated using the χ² test or Fisher exact test for categorical variables and the Mann–Whitney U test for continuous variables. N staging and LVI were classified as negative (–) or positive (+)

### Interobserver consensus of IVIM parameters and ADC

Both delineation strategies demonstrated high interobserver reproducibility. The ICC was 0.97 (95% CI: 0.96–0.98) for maximum-diameter delineation and 0.99 (95% CI: 0.99–0.99) for whole-volume delineation. For ADC metrics, whole-volume analysis showed better agreement for mean and standard deviation (ICC = 0.73), whereas maximum and minimum ADC values were more reproducible on the maximum-diameter slice (ICC = 0.84 and 0.88). For IVIM-derived parameters (D, D*, and f), whole-volume delineation provided consistently higher interobserver agreement, particularly for mean and standard deviation values (all ICCs > 0.85; Appendix Table S1).

### Comparison of IVIM parameters according to OS and RFS outcomes

In analyses limited to IVIM parameters with good interobserver agreement (ICC > 0.70), perfusion-related parameters showed significant differences between outcome groups. For maximum-diameter measurements (Appendix Table S2), f (mean) was significantly lower in deceased patients compared with survivors (*P* = 0.021) and also differed between recurrence and non-recurrence groups (*P* = 0.036). For whole-volume measurements (Appendix Table S3), only the f (minimum) value differed significantly between alive and deceased patients (*P* = 0.017), while no parameters showed significant differences for RFS.

### OS prediction models

Univariable Cox regression analyses of clinical and pathological variables for predicting OS are summarized in Table 2. After stepwise Cox regression, N stage was retained in the clinical model as an independent predictor of OS, with an HR of 2.538 (95% CI: 1.239–5.199, *P* = 0.010).

**Table 2:** Univariable Cox regression analysis of clinicopathological variables for overall survival and recurrence-free survival

Parameter	Overall Survival		Recurrence-Free Survival	
	HR (95% CI)	*P*	HR (95% CI)	*P*
Gender	1.103 (0.477, 2.550)	0.819	2.068 (0.719, 5.952)	0.178
Age	1.018 (0.965, 1.074)	0.509	0.951 (0.902, 1.003)	0.067
Smoking	0.803 (0.399, 1.618)	0.540	1.551 (0.745, 3.228)	0.241
Drinking	0.962 (0.480, 1.929)	0.913	1.267 (0.611, 2.625)	0.525
Location				
Up	1 (Reference)		1 (Reference)	
Middle	1.003 (0.288, 3.498)	0.996	0.711 (0.236, 2.145)	0.545
Low	1.401 (0.405, 4.850)	0.594	0.538 (0.168, 1.720)	0.300
Volume (cm^3^)	1.000 (1.000, 1.001)	0.175	1.000 (1.000, 1.001)	0.364
N Staging (+)	2.538 (1.239, 5.199)	0.010	2.635 (1.253, 5.540)	0.011
T Staging (3-4a)	1.468 (0.732, 2.944)	0.279	2.756 (1.298, 5.849)	0.008
LVI (+)	1.118 (0.552, 2.264)	0.757	1.049 (0.495, 2.222)	0.901
Histological Differentiation	1.812 (0.981, 3.346)	0.058	3.065 (1.590, 5.905)	0.001

HR = hazard ratio; CI = confidence interval; LVI = lymphovascular invasion

Histological differentiation was analyzed as an ordinal variable to assess the trend effect, coded as 1 = well differentiated, 2 = moderately differentiated, and 3 = poorly differentiated. The reported HR represents the risk increase per one-grade decrease in differentiation

For IVIM-based models, IVIM1 included f (mean) and D* (SD), whereas IVIM2 retained f (mean) and D* (SD) after feature selection (details shown in Table 3).

**Table 3:** Stepwise multivariable Cox regression analysis of IVIM parameters for overall survival and recurrence-free survival

Endpoint	Measurement	Parameter	HR (95% CI)	*P* value
OS	Maximum-diameter	f (mean), per 1%	0.936 (0.896, 0.977)	0.003
		D* (SD), per 1000 μm²/s	0.943 (0.904, 0.983)	0.006
	Whole-volume	f (mean), per 1%	0.900 (0.839, 0.965)	0.003
		D* (SD), per 1000 μm²/s	0.922 (0.869, 0.977)	0.006
RFS	Maximum-diameter	ADC (minimum), per 100 μm²/s	1.099 (0.998, 1.209)	0.055
		f (mean), per 1%	0.928 (0.885, 0.983)	0.002
	Whole-volume	D (maximum), per 100 μm²/s	0.912 (0.816, 1.020)	0.102
		f (SD), per 1%	0.892 (0.814, 0.977)	0.014
		f (minimum), per 1%	0.866 (0.760, 0.987)	0.030

HRs are presented with 95% CIs and represent the change in hazard for each unit increase specified in the Parameter column. OS = overall survival; RFS = recurrence-free survival; ADC = apparent diffusion coefficient; D = true diffusion coefficient; D* = pseudo-diffusion coefficient; f = perfusion fraction; SD = standard deviation. Maximum-diameter refers to IVIM parameters measured on the slice with the largest tumor diameter, whereas whole-volume refers to parameters extracted from the the whole-tumor volume. P values were calculated using multivariable Cox proportional hazards regression. Only variables retained after stepwise selection are shown

**Fig. 3:**
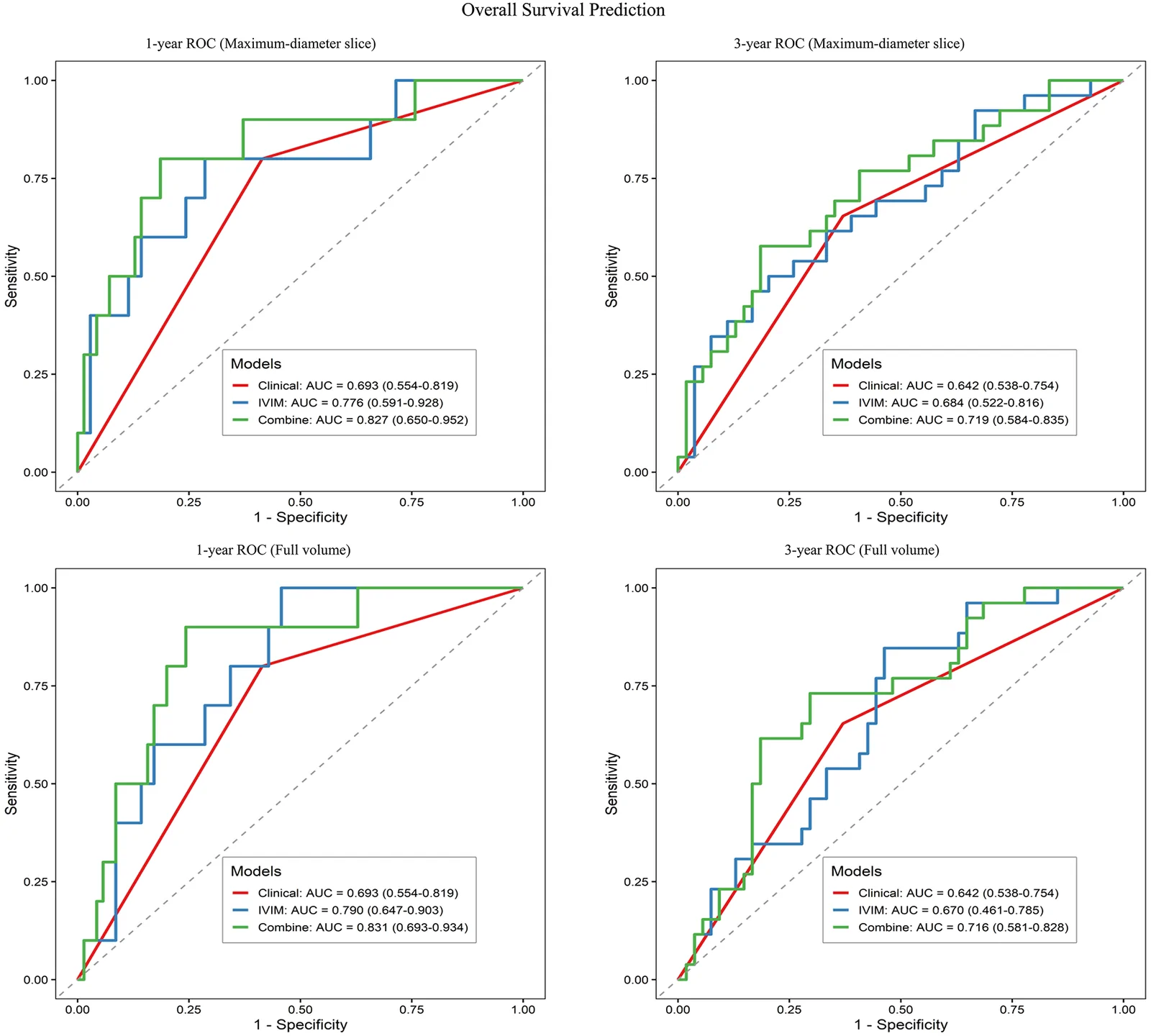
Time-dependent ROC curves for overall survival prediction. Time-dependent receiver operating characteristic (ROC) curves showing the predictive performance of the clinicopathological model, IVIM-based model, and combined model for 1-year and 3-year overall survival

Time-dependent ROC analysis demonstrated that IVIM-based models showed improved discriminative performance compared with the clinical model alone at both 1-year and 3-year OS (Fig. 3; Table 4). At 1 year, the combined models integrating clinical and IVIM parameters achieved the highest predictive performance, with AUCs of 0.827 for the Combined1 model and 0.831 for the Combined2 model. Similar trends were observed at 3 years, where combined models consistently outperformed individual clinical or IVIM models. After bootstrap optimism correction, the AUCs of all models were slightly reduced. Although Combined2 showed slightly higher AUC and sensitivity, its lower specificity and precision suggested more false-positive classifications; therefore, Combined1 was selected for subsequent analyses. Calibration curves of the IVIM–based combined model demonstrated good agreement between predicted and observed 3-year OS probabilities (Fig. S1).

**Table 4:** Predictive performance of clinical, IVIM-based, and combined models for 1-year and 3-year overall survival

Time point	Metric	Clinical	IVIM1	IVIM2	Combined1	Combined2
1-year	Accuracy	0.613	0.725	0.600	0.812	0.775
	Sensitivity	0.800	0.800	1.000	0.800	0.900
	Specificity	0.586	0.714	0.543	0.814	0.757
	Precision	0.216	0.286	0.238	0.381	0.346
	AUC (95% CI)	0.693 (0.554, 0.819)	0.776 (0.591, 0.928)	0.790 (0.647, 0.903)	0.827 (0.650, 0.952)	0.831 (0.693, 0.934)
	AUC (optimism-corrected)	0.691	0.765	0.782	0.815	0.815
3-year	Accuracy	0.637	0.700	0.637	0.738	0.713
	Sensitivity	0.654	0.500	0.846	0.577	0.731
	Specificity	0.630	0.796	0.537	0.815	0.704
	Precision	0.459	0.542	0.468	0.600	0.543
	AUC (95% CI)	0.642 (0.538, 0.754)	0.684 (0.522, 0.816)	0.670 (0.461, 0.785)	0.719 (0.584, 0.835)	0.716 (0.581, 0.828)
	AUC (optimism-corrected)	0.637	0.662	0.654	0.693	0.692

Apparent AUCs are reported with 95% confidence intervals. Optimism-corrected AUCs were calculated using bootstrap optimism correction and are reported as corrected point estimates. Model performance was evaluated at the 1-year and 3-year prediction time points using time-dependent receiver operating characteristic analysis. Clinical indicates the model constructed using clinical variables only; IVIM1 and IVIM2 indicate models constructed using IVIM-derived parameters from maximum-diameter and whole-volume analyses, respectively; Combined1 and Combined2 indicate models combining clinical variables with maximum-diameter and whole-volume IVIM-derived parameters, respectively. IVIM = intravoxel incoherent motion; AUC = area under the receiver operating characteristic curve; CI = confidence interval

### RFS prediction models

Univariable Cox regression analyses of clinical and pathological variables for predicting RFS are summarized in Table 2. After stepwise Cox regression, gender, T stage, pathological differentiation, and LVI were retained in the clinical model for RFS prediction. Among these factors, T stage (HR = 4.693, 95% CI: 1.839–11.976, *P* = 0.001) and pathological differentiation (HR = 2.721, 95% CI: 1.454–5.091, *P* = 0.002) were identified as strong predictors of recurrence, while LVI was inversely associated with RFS (HR = 0.267, 95% CI: 0.106–0.672, *P* = 0.005).

For IVIM-based models, IVIM1 included f (mean) and ADC (min), whereas the IVIM2 retained f (SD), f (min), and D (max) after feature selection (details shown in Table 3).

**Fig. 4:**
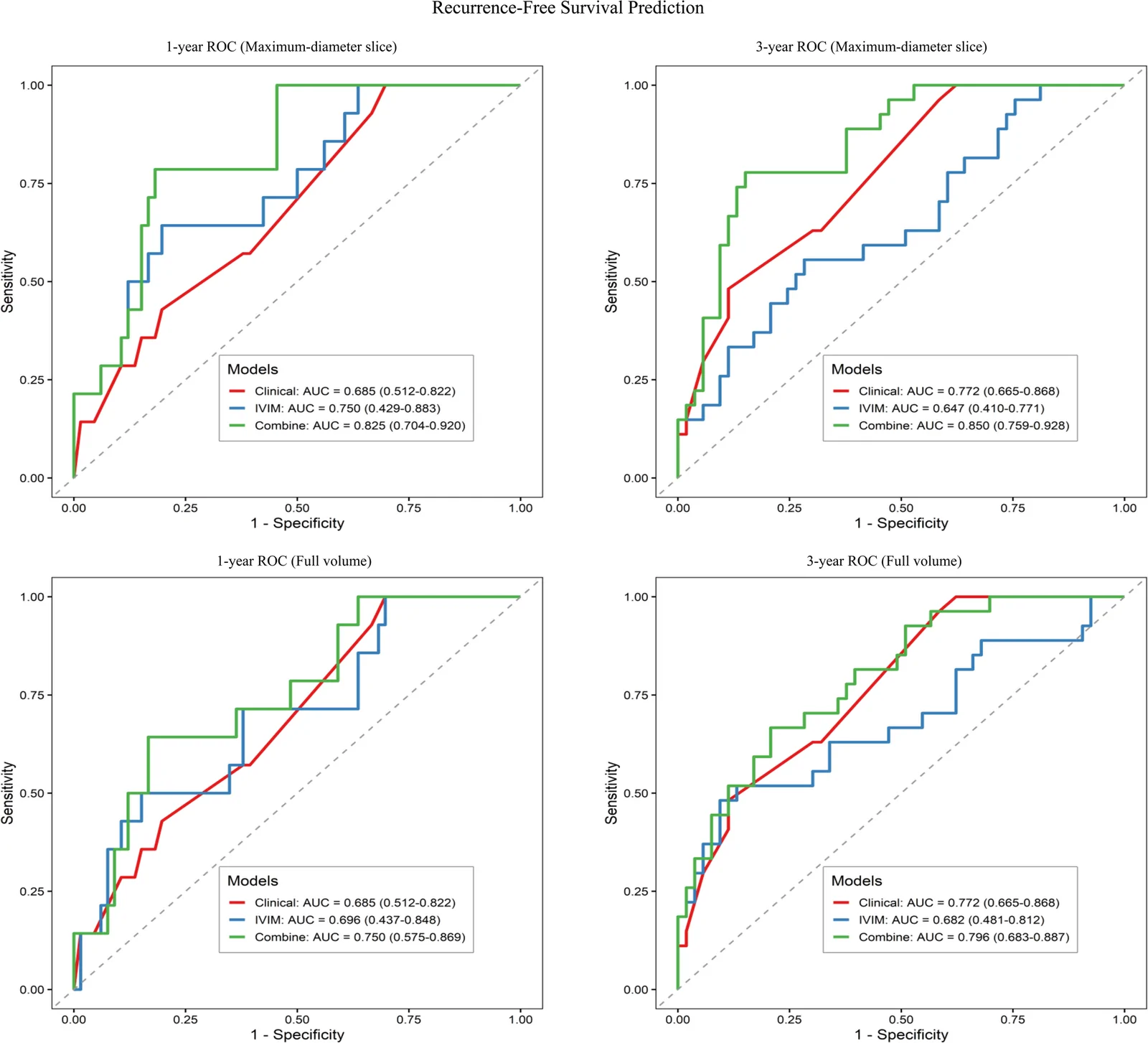
Time-dependent ROC curves for recurrence-free survival prediction. Time-dependent receiver operating characteristic (ROC) curves illustrating the predictive performance of the clinicopathological model, IVIM-based model, and combined model for 1-year and 3-year recurrence-free survival

Time-dependent ROC analysis showed that IVIM-based models outperformed the clinical model alone for both 1-year and 3-year RFS prediction (Fig. 4; Table 5). Among all models, Combined1 showed the highest AUCs at both 1 year and 3 years (0.825 and 0.850, respectively), and maintained corrected AUCs of 0.795 and 0.818 after bootstrap optimism correction. For RFS, calibration analysis showed that the optimal combined model based on IVIM parameters achieved satisfactory agreement at the 3-year time point (Fig. S2).

**Table 5:** Predictive performance of clinical, IVIM-based, and combined models for 1-year and 3-year recurrence-free survival

Time point	Metric	Clinical	IVIM1	IVIM2	Combined1	Combined2
1-year	Accuracy	0.425	0.774	0.787	0.812	0.800
	Sensitivity	1.000	0.643	0.500	0.786	0.643
	Specificity	0.303	0.803	0.848	0.818	0.833
	Precision	0.233	0.409	0.412	0.478	0.450
	AUC (95% CI)	0.685 (0.512, 0.822)	0.750 (0.429, 0.883)	0.696 (0.437, 0.848)	0.825 (0.704, 0.920)	0.750 (0.575, 0.869)
	AUC (optimism-corrected)	0.650	0.749	0.658	0.795	0.689
3-year	Accuracy	0.600	0.662	0.762	0.825	0.750
	Sensitivity	0.963	0.556	0.481	0.778	0.667
	Specificity	0.415	0.717	0.906	0.749	0.792
	Precision	0.456	0.500	0.722	0.724	0.621
	AUC (95% CI)	0.772 (0.665, 0.868)	0.647 (0.410, 0.771)	0.682 (0.481, 0.812)	0.850 (0.759, 0.928)	0.796 (0.683, 0.887)
	AUC (optimism-corrected)	0.737	0.636	0.644	0.818	0.731

Apparent AUCs are reported with 95% confidence intervals. Optimism-corrected AUCs were calculated using bootstrap optimism correction and are reported as corrected point estimates. Model performance was evaluated at the 1-year and 3-year prediction time points using time-dependent receiver operating characteristic analysis. Clinical indicates the model constructed using clinical variables only; IVIM1 and IVIM2 indicate models constructed using IVIM-derived parameters from maximum-diameter and whole-volume analyses, respectively; Combined1 and Combined2 indicate models combining clinical variables with maximum-diameter and whole-volume IVIM-derived parameters, respectively. IVIM = intravoxel incoherent motion; AUC = area under the receiver operating characteristic curve; CI = confidence interval

### Risk stratification for OS and RFS

Kaplan–Meier survival analysis based on the Combined model–derived risk scores demonstrated a clear separation between the high-risk and low-risk groups for both OS and RFS.

**Fig. 5:**
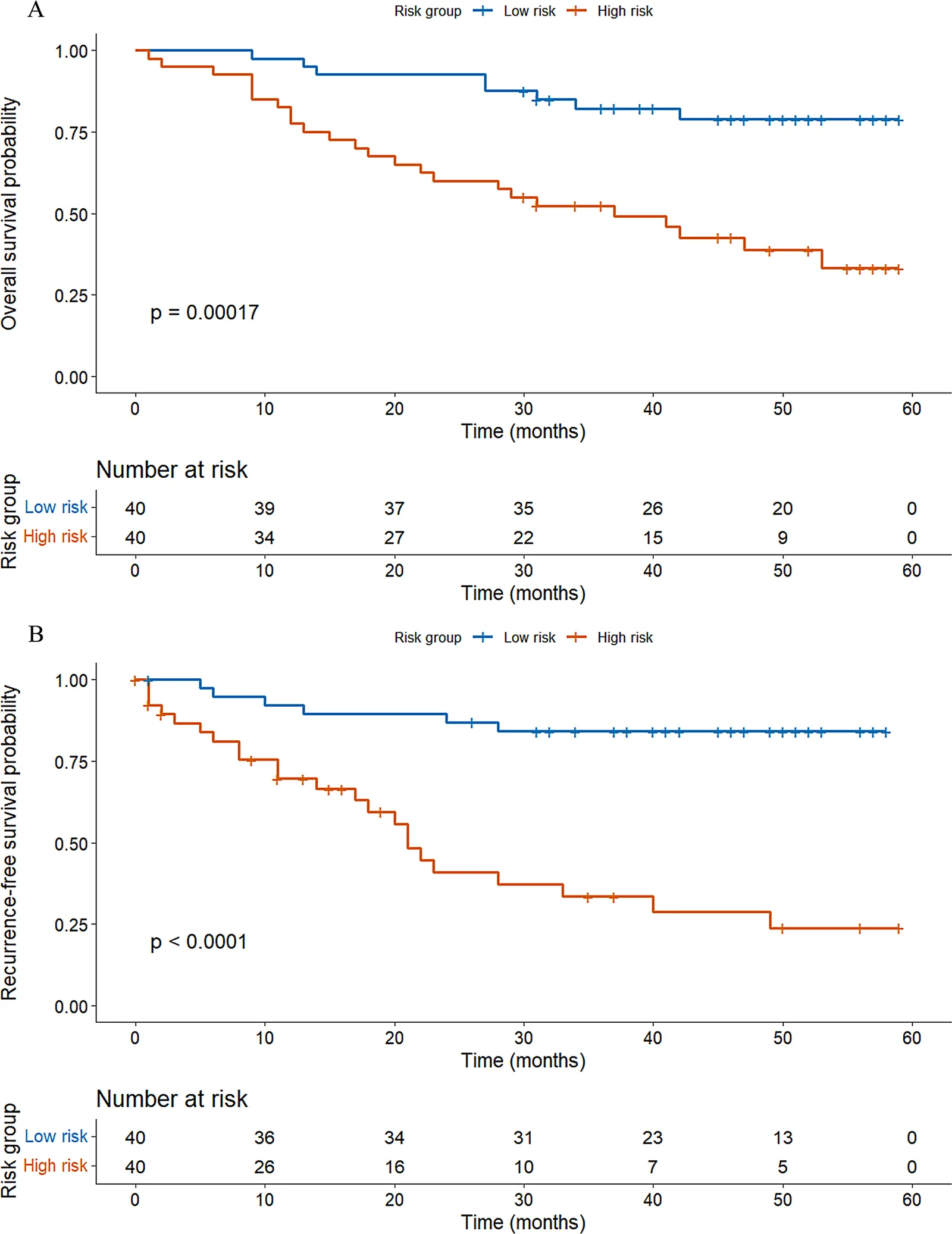
Kaplan–Meier survival curves stratified by predicted risk. Kaplan–Meier survival curves demonstrating risk stratification based on predicted probabilities from the combined maximum-diameter IVIM and clinicopathological models. Patients were divided into high- and low-risk groups according to median predicted risk. (**A**) Overall survival (OS), with a log-rank *P* < 0.001. (**B**) Recurrence-free survival (RFS), with a log-rank *P* < 0.001. The curves indicate that the models effectively discriminate patients with differing prognoses for both OS and RFS

For OS, patients in the high-risk group exhibited significantly poorer survival outcomes compared with those in the low-risk group. The Kaplan–Meier curves showed a distinct divergence between the two groups, and the log-rank test confirmed that the difference was statistically significant (*P* < 0.001) (Fig. 5A).

Similarly, for RFS, the high-risk group showed a significantly lower RFS rate than the low-risk group. Despite censoring patients who died without documented recurrence, the risk stratification remained robust, with well-separated survival curves and a statistically significant difference between groups as determined by the log-rank test (*P* < 0.001) (Fig. 5B).

## Discussion

In this study, we systematically compared the prognostic value of conventional pathological TN staging and IVIM-derived parameters for predicting OS and RFS in patients with esophageal cancer after surgical resection. The results demonstrated that IVIM parameters exhibited superior prognostic performance compared with traditional clinicopathological factors alone, particularly for OS prediction. Multivariable Cox regression analysis identified lymph node status as an independent prognostic factor, while selected IVIM-derived features, such as D* (SD), and f (mean), provided additional prognostic information beyond conventional staging. Furthermore, models incorporating maximum-diameter IVIM parameters achieved higher predictive accuracy than clinical models, and the combined models consistently showed the best performance for both OS and RFS.

The superior prognostic performance of IVIM-based models can be attributed to their ability to characterize intratumoral heterogeneity and microvascular alterations that are not captured by conventional TNM staging. While TNM classification primarily reflects macroscopic tumor extent and lymphatic involvement, IVIM-derived parameters provide quantitative information on diffusion restriction and perfusion-related microcirculation. In the present study, perfusion-related metrics derived from the f component consistently demonstrated prognostic relevance for both OS and RFS. Specifically, lower f values were associated with poorer survival and higher recurrence risk in both univariable and multivariable analyses, particularly for maximum-diameter slice measurements, suggesting that reduced microvascular perfusion may reflect dysfunctional angiogenesis, impaired oxygen delivery, and a hypoxic tumor microenvironment. Such conditions are known to promote aggressive tumor behavior, therapeutic resistance, and early recurrence [18].

Diffusion-related parameters also contributed complementary prognostic information. Measures reflecting diffusion heterogeneity, such as D* (SD), remained independently associated with outcomes in multivariable models, indicating that spatial variability in cellular density and stromal architecture may be more informative than mean diffusion values alone. Notably, parameters derived from the maximum-diameter slice often showed stronger associations with survival and recurrence than whole-volume metrics. This observation suggests that extreme biological features—such as regions with the poorest perfusion or most pronounced diffusion restriction—may disproportionately influence tumor progression and clinical outcomes. Together, these findings support the concept that IVIM parameters, particularly perfusion-related indices and heterogeneity measures, capture aggressive tumor phenotypes that are not adequately reflected by pathological staging and may explain heterogeneous prognoses among patients with similar TNM stages.

Previous studies have explored MRI and DWI in esophageal cancer for tumor characterization, treatment response assessment, and prognosis, but most have relied on single-parameter measurements or averaged diffusion values [12, 19, 20]. IVIM offers several advantages over conventional DWI by separating true diffusion from perfusion-related diffusion, thereby providing physiologically interpretable parameters related to tumor cellularity and microcirculatory perfusion [21, 22]. Compared with high-dimensional radiomics approaches, IVIM may be easier to interpret biologically because it yields a limited number of quantitative parameters with relatively direct physiological meaning. Our results build upon these findings by systematically evaluating multiple IVIM-derived parameters from both maximum-diameter and whole-volume measurements and demonstrating their added prognostic value, particularly when incorporated into combined models with pathological staging.

The inverse association between LVI and RFS in the multivariable model was unexpected and should be interpreted with caution. Because LVI was not significantly associated with RFS in univariable analysis, its apparent protective association after adjustment is unlikely to represent a true biological effect. This finding may be partly related to selection bias in this surgical cohort, as some patients who underwent IVIM-DWI were subsequently treated with neoadjuvant therapy and were not included in the final analysis; these patients may have had more aggressive tumors and a higher likelihood of LVI positivity. Residual confounding and model instability due to the limited number of recurrence events may also have contributed to this result.

These findings may have potential implications for individualized treatment stratification and postoperative surveillance in patients with ESCC. IVIM-derived parameters may help identify patients with biologically aggressive tumors who are at increased risk of recurrence or death despite apparently resectable disease, thereby providing complementary information for multidisciplinary evaluation and postoperative risk assessment [23]. After surgery, combined models incorporating IVIM parameters and pathological staging may help refine recurrence risk assessment beyond TNM staging alone. Nevertheless, although multicenter studies of MRI-derived parameters have been increasingly performed [24, 25], further standardization of IVIM acquisition and analysis workflows remains necessary to ensure reproducible parameter measurements across institutions. Importantly, the potential implications of IVIM-based models for treatment stratification and surveillance strategies should be considered hypothesis-generating until confirmed by prospective multicenter external validation.

This study has several limitations. First, the reproducibility of IVIM-derived parameters remains an important concern. Although overall tumor delineation showed high interobserver reproducibility, several individual IVIM-derived metrics demonstrated only moderate ICCs, indicating that some parameters may be vulnerable to measurement variability. Because IVIM-based models rely on selected quantitative parameters, moderate reproducibility may influence feature selection, risk-score calculation, and patient risk classification. IVIM parameters may also vary according to acquisition protocols, b-value distribution, signal-to-noise ratio, motion artifacts, fitting algorithms, and segmentation methods, which may reduce predictor stability and limit model generalizability across scanners, readers, and institutions. Standardized acquisition protocols, harmonized post-processing pipelines, consistent segmentation strategies, and multicenter external validation are therefore needed before clinical translation. Second, the final cohort included only patients who underwent both preoperative IVIM-DWI and surgical resection without neoadjuvant therapy; therefore, selection bias may have been introduced, particularly because some patients who underwent IVIM-DWI were subsequently treated with neoadjuvant therapy and excluded from the analysis. Third, the retrospective single-center design and relatively small sample size may limit the robustness and generalizability of the findings. Although bootstrap optimism correction was performed, the slightly lower corrected AUCs compared with the apparent AUCs indicate that some degree of optimism may remain in model performance estimates. Therefore, larger cohorts and external validation are needed in future studies.

## Conclusion

In conclusion, IVIM-derived MRI parameters offer valuable prognostic information in esophageal cancer, capturing tumor microstructural and perfusion characteristics that complement conventional pathological staging. Integrating IVIM features with clinicopathological factors may improve postoperative risk stratification and provide a basis for future individualized management strategies.

## Supplementary Information

Below is the link to the electronic supplementary material.

Supplementary Material 1

Supplementary Material 2: Figure S1: Calibration curves of the combined models for 1-year and 3-year overall survival prediction.Calibration curves show the agreement between predicted and observed overall survival (OS) probabilities at 1 year and 3 years. The dashed diagonal line represents perfect calibration. The green and orange curves represent the combined models based on maximum-diameter IVIM (IVIM1) parameters and whole-volume (IVIM2) parameters, respectively.

Supplementary Material 3: Figure S2: Calibration curves of the combined models for 1-year and 3-year recurrence-free survival prediction.Calibration curves show the agreement between predicted and observed recurrence-free survival (RFS) probabilities at 1 year and 3 years. The dashed diagonal line represents perfect calibration. The green and orange curves represent the combined models based on maximum-diameter IVIM (IVIM1) parameters and whole-volume IVIM (IVIM2) parameters, respectively.

## Data Availability

The datasets generated during and/or analysed during the current study are available from the corresponding author on reasonable request.

## References

[1] Sung H, Ferlay J, Siegel RL, Laversanne M, Soerjomataram I, Jemal A, Bray F. Global Cancer Statistics 2020: GLOBOCAN Estimates of Incidence and Mortality Worldwide for 36 Cancers in 185 Countries. CA Cancer J Clin. 2021;71(3):209–49. 33538338 10.3322/caac.21660

[2] Rice TW, Ishwaran H, Ferguson MK, Blackstone EH, Goldstraw P. Cancer of the Esophagus and Esophagogastric Junction: An Eighth Edition Staging Primer. J Thorac Oncol. 2017;12(1):36–42. 27810391 10.1016/j.jtho.2016.10.016 PMC5591443

[3] Li J, Zhang H, Bei T, Wang Y, Ma F, Wang S, Li H, Qu J. Advanced diffusion-weighted MRI models for preoperative prediction of lymph node metastasis in resectable gastric cancer. Abdom Radiol. 2024;50(3):1057–68. 10.1007/s00261-024-04559-3 39254709

[4] Liu Y, Wang Y, Hu X, Wang X, Xue L, Pang Q, Zhang H, Ma Z, Deng H, Yang Z, et al. Multimodality deep learning radiomics predicts pathological response after neoadjuvant chemoradiotherapy for esophageal squamous cell carcinoma. Insights Imaging. 2024;15(1). 10.1186/s13244-024-01851-0 PMC11568088 39546168

[5] Meyer HJ, Höhn AK, Woidacki K, Andric M, Powerski M, Pech M, Surov A. Associations between IVIM histogram parameters and histopathology in rectal cancer. Magn Reson Imaging. 2021;77:21–7. 33316358 10.1016/j.mri.2020.12.008

[6] Song T, Yao Q, Qu J, Zhang H, Zhao Y, Qin J, Feng W, Zhang S, Han X, Wang S, et al. The value of intravoxel incoherent motion diffusion-weighted imaging in predicting the pathologic response to neoadjuvant chemotherapy in locally advanced esophageal squamous cell carcinoma. Eur Radiol. 2020;31(3):1391–400. 32901300 10.1007/s00330-020-07248-z

[7] Wang Y, Bai G, Guo L, Chen W. Associations between apparent diffusion coefficient value with pathological type, histologic grade, and presence of lymph node metastases of esophageal carcinoma. Technol Cancer Res Treat. 2019;18. 10.1177/1533033819892254 PMC6886268 31782340

[8] Le Bihan D. What can we see with IVIM MRI? Neuroimage. 2019;187:56-67. 10.1016/j.neuroimage.2017.12.062 29277647

[9] Zhu S, Wei Y, Gao F, Li L, Liu Y, Huang Z, Tang H, Zheng D, Wei X, Sun T, et al. Esophageal carcinoma: Intravoxel incoherent motion diffusion-weighted MRI parameters and histopathological correlations. J Magn Reson Imaging. 2018;49(1):253–61. 29734492 10.1002/jmri.26172

[10] Zheng H, Ren W, Pan X, Zhang Q, Liu B, Liu S, He J, Zhou Z. Role of intravoxel incoherent motion MRI in early assessment of the response of esophageal squamous cell carcinoma to chemoradiotherapy: A pilot study. J Magn Reson Imaging. 2018;48(2):349–58. 29297204 10.1002/jmri.25934

[11] Heethuis SE, Goense L, van Rossum PSN, Borggreve AS, Mook S, Voncken FEM, Bartels-Rutten A, Aleman BMP, van Hillegersberg R, Ruurda JP, et al. DW-MRI and DCE-MRI are of complementary value in predicting pathologic response to neoadjuvant chemoradiotherapy for esophageal cancer. Acta Oncol. 2018;57(9):1201–8. 29781342 10.1080/0284186X.2018.1473637

[12] Mizumachi R, Hayano K, Hirata A, Ohira G, Imanishi S, Tochigi T, Isozaki T, Kurata Y, Ikeda Y, Urahama R, et al. Development of imaging biomarker for esophageal cancer using intravoxel incoherent motion MRI. Esophagus. 2021;18(4):844–50. 34019200 10.1007/s10388-021-00851-z

[13] Liu N, Yang X, Lei L, Pan K, Liu Q, Huang X. Intravoxel incoherent motion model in differentiating the pathological grades of esophageal carcinoma: comparison of mono-exponential and bi-exponential fit model. Front Oncol. 2021;11. 10.3389/fonc.2021.625891 PMC8071935 33912449

[14] Shao T, Liu S, Qin J, Zhang H, Wang Z, Jiang Z, Li H, Zhang Y, Qiao J, Feng W, et al. Intravoxel incoherent motion diffusion-weighted imaging in evaluating preoperative staging of esophageal squamous cell carcinoma: evaluation of preoperative stage of primary tumour and prediction of lymph node metastases from esophageal cancer using IVIM: a prospective study. Cancer Imaging. 2024;24(1). 10.1186/s40644-024-00765-w PMC11363402 39210470

[15] Du G, Chen L, Wen B, Lu Y, Xia F, Liu Q, Shen W. Deep learning-based prediction of tumor aggressiveness in RCC using multiparametric MRI: a pilot study. Int Urol Nephrol. 2024;57(5):1365–79. 39671158 10.1007/s11255-024-04300-5 PMC12003571

[16] Park JE, Park SY, Kim HJ, Kim HS. Reproducibility and generalizability in radiomics modeling: possible strategies in radiologic and statistical perspectives. Korean J Radiol. 2019;20(7). 10.3348/kjr.2018.0070 PMC6609433 31270976

[17] Feng F, Kong L, Yang C, Wang Y, Chen J, Zhou J, Hu Y. Preoperative esophageal cancer staging assessment based on intravoxel incoherent motion and apparent diffusion coefficient: a comparative study of maximum-diameter slice region of interest and whole volume of interest analysis. BMC Med Imaging. 2025;25(1):444. 41188750 10.1186/s12880-025-01973-x PMC12584429

[18] Gaustad JV, Hauge A, Wegner CS, Simonsen TG, Lund KV, Hansen LMK, Rofstad EK. DCE-MRI of tumor hypoxia and hypoxia-associated aggressiveness. Cancers (Basel). 2020;12(7). 10.3390/cancers12071979 PMC7409330 32698525

[19] Bai B, Cui L, Chu F, Wang Z, Zhao K, Wang S, Wang S, Yan X, Wang M, Kamel IR, et al. Multiple diffusion models for predicting pathologic response of esophageal squamous cell carcinoma to neoadjuvant chemotherapy. Abdom Radiol (NY). 2024;49(12):4216–26. 38954001 10.1007/s00261-024-04474-7

[20] Song T, Lu S, Qu J, Zhang H, Wang Z, Jia Z, Li H, Zhao Y, Qin J, Feng W, et al. Intravoxel incoherent motion diffusion-weighted imaging in evaluating preoperative staging of esophageal squamous cell carcinoma: Evaluation of preoperative stage of primary tumour and prediction of lymph node metastases from esophageal cancer using IVIM: a prospective study. Cancer Imaging. 2024;24(1):116. 39210470 10.1186/s40644-024-00765-w PMC11363402

[21] Szubert-Franczak AE, Naduk-Ostrowska M, Pasicz K, Podgórska J, Skrzyński W, Cieszanowski A. Intravoxel incoherent motion magnetic resonance imaging: basic principles and clinical applications. Pol J Radiol. 2020;85:e624–35. 33376564 10.5114/pjr.2020.101476 PMC7757509

[22] Fan Y, Wang H, Wang S, Liu X, Yang X, Li L, Zhang Y, Jiang Y, Xu L, Li M, et al. The prognostic value of intra- and peritumoral habitat analysis in nasopharyngeal carcinoma: combining IVIM and ASL. Eur Radiol. 2025. 10.1007/s00330-025-12121-y 41417122

[23] van Houdt PJ, Václavů L, Sourbron S, Shalom ES, Federau C, Iima M, Liu MM, Knutsson L, Wirestam R, Günther M, et al. ESR essentials: perfusion MRI—practice recommendations by the European Society for Magnetic Resonance in Medicine and Biology. Eur Radiol. 2026. 10.1007/s00330-025-12306-5 PMC13212675 41591472

[24] Yunsong L, Yi W, Xin W, Liyan X, Huan Z, Zeliang M, Heping D, Zhaoyang Y, Xujie S, Yu M, et al. MR radiomics predicts pathological complete response of esophageal squamous cell carcinoma after neoadjuvant chemoradiotherapy: a multicenter study. Cancer Imaging. 2024;24(1). 10.1186/s40644-024-00659-x PMC10804642 38263134

[25] Tornari M, Rossi F, Moretti C, Giordano A, Ricci S, Verdi S, Zanetti M, Iacobelli LJ, Giusti C, Conti M, et al. The prognostic role of MRI-based radiomics in tongue carcinoma: a multicentric validation study. Radiol Med. 2024;129(9). 10.1007/s11547-024-01859-y PMC11379741 39096355

