# Supplementary material for: Prognostic value of IVIM-DWI parameters for overall and recurrence-free survival in resected esophageal squamous cell carcinoma
Source: Cancer Imaging. 2026 Jun 25;26:124. doi: 10.1186/s40644-026-01077-x (PMC13560107; doi:10.1186/s40644-026-01077-x)
Supplement: Supplementary file 1 [file 40644_2026_1077_MOESM1_ESM.docx]

Table S1: ICC Analysis of Parameters at the Largest Cross-Sectional Area and Whole Volume

| Parameter | Maximum-diameter | | |  | Full volume | | |
| --- | --- | --- | --- | --- | --- | --- | --- |
|  | OB1 | OB2 | ICC (CI 95%) |  | OB1 | OB2 | ICC (CI 95%) |
| Volume (cm^3^) | 1.33 (0.79, 1.80) | 1.78 (1.37, 2.76) | 0.97 (0.96, 0.98) |  | 6.09 (2.72, 12.37) | 6.10 (2.90, 12.62) | 0.99 (0.99, 0.99) |
| ADC |  |  |  |  |  |  |  |
| mean (um^2^/s) | 1806.90 (1547.10, 2016.60) | 1829.00 (1653.60, 2063.10) | 0.14 (0, 0.45) |  | 1838.40 (1644.20,2002.10) | 1784.10 (1593.80, 2036.80) | 0.73 (0.58, 0.83) |
| SD (um^2^/s) | 256.10 (202.20, 307.30) | 257.80 (212.10,319.50) | 0.44 (0.12, 0.64) |  | 249.90 (203.30,300.90) | 234.00 (198.60, 304.70) | 0.73 (0.59, 0.83) |
| minimum (um^2^/s) | 1142.66±469.92 | 1180.02±460.10 | 0.88 (0.81, 0.92) |  | 948.00 (649.10,1198.30) | 939.90 (663.70, 1290.90) | 0.22 (0, 0.50) |
| maximum (um^2^/s) | 2398.60 (2234.70,2564.30) | 2420.40 (2275.10,2606.20) | 0.84 (0.75, 0.90) |  | 2662.20 (2503.40, 2821.60) | 2524.10 (2351.30, 2738.10) | 0.41 (0.08, 0.62) |
| D |  |  |  |  |  |  |  |
| mean (um^2^/s) | 1960.50 ± 453.30 | 1970.30 ± 437.70 | 0.97 (0.95, 0.98) |  | 1972.90 (1778.40,2233.20) | 1949.50 (1736.30, 2220.90) | 0.92 (0.88, 0.95) |
| SD (um^2^/s) | 319.00 (257.20, 379.10) | 326.40 (254.30,400.30) | 0.91 (0.86, 0.94) |  | 325.30 (258.40,357.50) | 301.60 (257.80, 350.80) | 0.85 (0.78, 0.91) |
| minimum (um^2^/s) | 1247.10 (969.60, 1564.50) | 1264.40 (973.30, 1484.40) | 0.90 (0.84, 0.94) |  | 979.30 (573.70,1262.00) | 861.50 (546.40, 1269.00) | 0.56 (0.31, 0.72) |
| maximum (um^2^/s) | 2731.60 (2481.40, 3067.90) | 2813.40 (2521.40, 3058.60) | 0.02 (0, 0.37) |  | 3103.40 (2870.30,3311.90) | 3025.50 (2759.00, 3324.50) | 0.72 (0.56, 0.82) |
| 𝑓 |  |  |  |  |  |  |  |
| mean | 0.28 (0.23, 0.34) | 0.27 (0.21, 0.33) | 0.93 (0.88, 0.95) |  | 0.30 (0.30,0.30) | 0.30 (0.30,0.30) | 0.93 (0.90, 0.96) |
| SD | 0.10 (0.10, 0.20) | 0.10 (0.10, 0.20) | 0.86 (0.78, 0.91) |  | 0.16±0.05 | 0.16±0.05 | 0.88 (0.82, 0.93) |
| minimum | 0.00 (0.00,0.10) | 0.00 (0.00,0.10) | 0.00 (0, 0.36) |  | 0.00 (0.00,0.00) | 0.00 (0.00,0.00) | 0.88 (0.81, 0.92) |
| maximum | 0.80 (0.60, 1.00) | 1.00 (0.60, 1.00) | 0.00 (0, 0.36) |  | 1.00 (1.00, 1.00) | 1.00 (1.00, 1.00) | 0.89 (0.83, 0.93) |
| D* |  |  |  |  |  |  |  |
| mean (um^2^/s) | 36087.60 (25706.60, 54368.20) | 33115.10 (24376.70, 48433.00) | 0.81 (0.71, 0.88) |  | 38367.70 (32050.90,46875.60) | 35901.00 (29302.70,44905.10) | 0.90 (0.84, 0.93) |
| SD (um^2^/s) | 28729.72±10973.63 | 28828.03±10150.94 | 0.77 (0.65, 0.85) |  | 28804.51±6910.66 | 28356.96±7139.25 | 0.91 (0.86, 0.94) |
| minimum (um^2^/s) | 1198.00 (160.30, 2795.40) | 1084.50 (616.00,2212.80) | 0.57 (0.33, 0.72) |  | 0 (0,1034.70) | 19.80 (0,952.70) | 0.89 (0.82, 0.93) |
| maximum (um^2^/s) | 100000.00 (100000.00,100000.00) | 100000.00 (100000.00,100000.00) | 0.07 (0, 0.40) |  | 100000.00 (100000.00,100000.00) | 100000.00 (100000.00,100000.00) | 0.34 (0, 0.58) |

ICC = intra-class correlation coefficients; SD = standard deviation. ADC = apparent diffusion coefficient; SD = standard deviation; D = true diffusion coefficient; D* = pseudo-diffusion coefficient; f = perfusion fraction. Maximum-diameter refers to IVIM parameters measured on the slice with the largest tumor diameter, whereas full volume refers to parameters extracted from the whole-tumor volume. Due to the slice thickness of MR images, the measurements obtained, even from a single slice, represent volumetric data rather than purely area data.

Table S2: Comparison of IVIM parameters derived from maximum-diameter measurements between different OS and RFS outcome groups (parameters with ICC > 0.75).

| Parameter | OS | | |  | RFS | | |
| --- | --- | --- | --- | --- | --- | --- | --- |
|  | Alive (n = 48) | Death (n = 32) | *P* |  | No recurrence (n =29) | Recurrence (n = 51) | *P* |
| ADC |  |  |  |  |  |  |  |
| minimum (um^2^/s) | 1095.2 (798.33-1484.21) | 1257.86 (1062.1-1415.13) | 0.371 |  | 1238.86 (1071.91-1373.92) | 1118.49 (817.94-1447.26) | 0.548 |
| maximum (um^2^/s) | 2396.63 (2228.52-2543.74) | 2441.56 (2285.38-2620.57) | 0.335 |  | 2361.28 (2234.64-2500.18) | 2427.9 (2284.52-2604.71) | 0.484 |
| D |  |  |  |  |  |  |  |
| mean (um^2^/s) | 1969 (1757.62-2346.67) | 1873.36 (1676.85-2145.96) | 0.238 |  | 1889.09 (1680.16-2104.56) | 1980.84 (1747.03-2367.85) | 0.147 |
| SD (um^2^/s) | 304.07 (263.6-379.33) | 340.94 (265.67-397.47) | 0.219 |  | 287.82 (253.98-355.1) | 351.57 (270.98-396.48) | 0.389 |
| minimum (um^2^/s) | 1212.77 (920.59-1615.71) | 1162.72 (948.02-1450.9) | 0.568 |  | 1165.25 (891.53-1445.51) | 1211.8 (944.69-1593.75) | 0.624 |
| f |  |  |  |  |  |  |  |
| mean | 0.29 (0.24-0.38) | 0.25 (0.2-0.31) | 0.021 |  | 0.27 (0.18-0.31) | 0.28 (0.23-0.38) | 0.036 |
| SD | 0.15 (0.1-0.21) | 0.12 (0.1-0.19) | 0.636 |  | 0.15 (0.09-0.2) | 0.12 (0.1-0.2) | 0.756 |
| D* |  |  |  |  |  |  |  |
| mean (um^2^/s) | 40942.4 (30300.19-47775.08) | 29558.65 (21802.91-49201.72) | 0.085 |  | 35484.65 (25316.85-45119.9) | 36679.7 (27856.78-52253.07) | 0.652 |
| SD (um^2^/s) | 30577.15 (18817.39-38951.02) | 25041.25 (21805.34-32450.61) | 0.16 |  | 32409.1 (23593.35-37063.8) | 25245.6 (21435.97-36021.88) | 0.2 |

OS = overall survival; RFS = recurrence-free survival; ADC = apparent diffusion coefficient; D = true diffusion coefficient; SD = standard deviation; D* = pseudo-diffusion coefficient; f = perfusion fraction. P values were calculated using the Mann–Whitney U test.

Data are presented as median (interquartile range). Only IVIM-derived parameters with good interobserver agreement (ICC > 0.75) were included.

Table S3: Comparison of IVIM parameters derived from full volume measurements between different OS and RFS outcome groups (parameters with ICC > 0.75).

| Parameter | OS | | |  | RFS | | |
| --- | --- | --- | --- | --- | --- | --- | --- |
|  | Alive (n = 48) | Death (n = 32) | *P* |  | No recurrence (n =29) | Recurrence (n = 51) | *P* |
| ADC |  |  |  |  |  |  |  |
| mean (um^2^/s) | 1812.51 (1620.04-2013.58) | 1772.29 (1607.62-1927.32) | 0.302 |  | 1762.53 (1625.61-1945.82) | 1809.22 (1606.87-2019.81) | 0.275 |
| SD (um^2^/s) | 236.86 (190.25-297.96) | 250.39 (209.7-298.78) | 0.461 |  | 240.24 (195.55-283.94) | 243.02 (202.33-308.06) | 0.779 |
| D |  |  |  |  |  |  |  |
| mean (um^2^/s) | 2056.18 (1720.62-2294.06) | 1901.43 (1785.27-2142.74) | 0.215 |  | 1894.38 (1771.9-2120.8) | 2052.02 (1732.17-2280.15) | 0.136 |
| SD (um^2^/s) | 298.59 (259.37-351.5) | 317.53 (288.54-354.81) | 0.288 |  | 299.34 (275.43-348.87) | 315.13 (272.05-355.05) | 0.952 |
| minimum (um^2^/s) | 967.36 (528.04-1418.1) | 802.04 (506.79-1082.01) | 0.214 |  | 826.96 (577-1040.1) | 1046.56 (517.78-1370.4) | 0.447 |
| maximum (um^2^/s) | 3052.74 (2872.49-3237.11) | 3010.02 (2834.5-3275.59) | 0.895 |  | 3030.74 (2828.54-3171.57) | 3052.13 (2887.35-3317.94) | 0.401 |
| f |  |  |  |  |  |  |  |
| mean | 0.3 (0.27-0.36) | 0.28 (0.24-0.31) | 0.056 |  | 0.29 (0.24-0.32) | 0.29 (0.26-0.35) | 0.158 |
| SD | 0.16 (0.12-0.2) | 0.15 (0.12-0.19) | 0.736 |  | 0.13 (0.12-0.18) | 0.16 (0.13-0.19) | 0.250 |
| minimum | 0 (0-0.02) | 0 (0-0) | 0.017 |  | 0 (0-0) | 0 (0-0) | 0.237 |
| maximum | 1 (0.91-1) | 1 (1-1) | 0.396 |  | 1 (1-1) | 1 (0.96-1) | 0.884 |
| D* |  |  |  |  |  |  |  |
| mean (um^2^/s) | 38562.2 (33245.24-46582.93) | 36244.68 (30901.65-43702.71) | 0.302 |  | 36228.4 (31232.65-43483) | 38233.1 (33187.88-47020.07) | 0.193 |
| SD (um^2^/s) | 28915.65 (24819.85-35006.22) | 29073.32 (25952.09-31415.69) | 0.736 |  | 29138.55 (26635.45-31788.45) | 28253.55 (23310.92-31604.68) | 0.555 |
| minimum (um^2^/s) | 69.24 (0-994.42) | 186.46 (0-523.17) | 0.749 |  | 223.38 (0-661.18) | 0 (0-959.13) | 0.607 |

OS = overall survival; RFS = recurrence-free survival; ADC = apparent diffusion coefficient; SD = standard deviation; D = true diffusion coefficient; D* = pseudo-diffusion coefficient; f = perfusion fraction. P values were calculated using the Mann–Whitney U test.

Data are presented as median (interquartile range). Only IVIM-derived parameters with good interobserver agreement (ICC > 0.75) were included.

Table S4. Univariate Cox regression analysis of IVIM parameters for overall survival and recurrence-free survival.

| Parameter | OS | | | | |  | RFS | | | | |
| --- | --- | --- | --- | --- | --- | --- | --- | --- | --- | --- | --- |
|  | Maximum-diameter | |  | Full volume | |  | Maximum-diameter | |  | Full volume | |
|  | HR (95%CI） | P |  | HR (95%CI） | P |  | HR (95%CI） | P |  | HR (95%CI） | P |
| ADC |  |  |  |  |  |  |  |  |  |  |  |
| mean (100um^2^/s) | - | - |  | 0.969 (0.888, 1.057) | 0.475 |  | - | - |  | 0.940 (0.865, 1.021) | 0.143 |
| SD (10um^2^/s) | - | - |  | 1.011 (0.975, 1.047) | 0.558 |  | - | - |  | 1.004 (0.963, 1.047) | 0.856 |
| minimum (100um^2^/s) | 1.010 (0.940, 1.085) | 0.785 |  | - | - |  | 1.012 (0.941, 1.089) | 0.743 |  | - | - |
| maximum (100um^2^/s) | 1.046 (0.957, 1.142) | 0.323 |  | - | - |  | 1.006 (0.915, 1.105) | 0.902 |  | - | - |
| D |  |  |  |  |  |  |  |  |  |  |  |
| mean (100um^2^/s) | 0.973 (0.905, 1.045) | 0.450 |  | 0.966 (0.898, 1.039) | 0.356 |  | 0.950 (0.884, 1.021) | 0.161 |  | 0.937 (0.872, 1.007) | 0.103 |
| SD (10um^2^/s) | 1.011 (0.988, 1.034) | 0.366 |  | 1.014 (0.980, 1.049) | 0.432 |  | 1.010 (0.982, 1.039) | 0.471 |  | 1.014 (0.974, 1.056) | 0.495 |
| minimum (100um^2^/s) | 0.980 (0.922, 1.043) | 0.529 |  | 0.954 (0.895, 1.017) | 0.147 |  | 0.976 (0.917, 1.040) | 0.454 |  | 0.958 (0.898, 1.022) | 0.190 |
| maximum (100um^2^/s) | - | - |  | 1.027 (0.930, 1.134) | 0.601 |  | - | - |  | 0.943 (0.851, 1.044) | 0.258 |
| f |  |  |  |  |  |  |  |  |  |  |  |
| mean (%) | 0.959 (0.922, 0.998) | 0.040 |  | 0.951 (0.907, 0.998) | 0.042 |  | 0.945 (0.905, 0.988) | 0.012 |  | 0.952 (0.905, 1.000) | 0.052 |
| SD (%) | 0.990 (0.941, 1.041) | 0.687 |  | 0.982 (0.914, 1.054) | 0.610 |  | 1.017 (0.964, 1.072) | 0.538 |  | 0.968 (0.897, 1.044) | 0.401 |
| minimum (%) | - | 0.057 |  | 0.926 (0.837, 1.025) | 0.138 |  | - | - |  | 0.926 (0.838, 1.024) | 0.132 |
| maximum (%) | - | - |  | 1.005 (0.971, 1.040) | 0.788 |  | - | - |  | 1.011 (0.975, 1.048) | 0.556 |
| D* |  |  |  |  |  |  |  |  |  |  |  |
| mean (1000um^2^/s) | 0.990 (0.969, 1.012) | 0.379 |  | 0.987 (0.960, 1.015) | 0.356 |  | 0.993 (0.970, 1.017) | 0.581 |  | 0.977 (0.946, 1.009) | 0.165 |
| SD (1000um^2^/s) | 0.974 (0.939, 1.011) | 0.161 |  | 0.987 (0.941, 1.035) | 0.592 |  | 1.022 (0.984, 1.062) | 0.264 |  | 1.015 (0.963, 1.070) | 0.584 |
| minimum (um^2^/s) | - | - |  | 0.982 (0.957, 1.008) | 0.183 |  | - | - |  | 0.992 (0.976, 1.009) | 0.358 |
| maximum (um^2^/s) | - | - |  | - | - |  | - | - |  | - | - |

HRs are presented with 95% CIs and correspond to each unit increase in the rescaled parameter shown in the Parameter column. For instance, “100 μm²/s” indicates that the HR is reported per 100 μm²/s increase, whereas “%” indicates that the HR is reported per 1% increase. OS = overall survival; RFS = recurrence-free survival; ADC = apparent diffusion coefficient; SD, standard deviation; D = true diffusion coefficient; D* = pseudo-diffusion coefficient; f = perfusion fraction. Maximum-diameter refers to IVIM parameters measured on the slice with the largest tumor diameter, whereas full volume refers to parameters extracted from the whole-tumor volume. “–” indicates that the parameter was not included in the corresponding analysis. P values were calculated using univariate Cox proportional hazards regression.
